# English as a Foreign Language Teachers’ Critical Thinking Ability and L2 Students’ Classroom Engagement

**DOI:** 10.3389/fpsyg.2021.773138

**Published:** 2021-11-12

**Authors:** Ziguang Yan

**Affiliations:** School of Foreign Studies, Hebei University, Baoding, China

**Keywords:** critical thinking, classroom engagement, foreign language learning, EFL classroom, EFT teacher

## Abstract

Critical thinking has been the focus of many studies considering the educational and social contexts. However, English as a foreign language (EFL) context is the one in which studies about critical thinking and its link to classroom engagement have not been carried out as much as expected. Hence, this study investigated to understand the association between EFL teachers’ critical thinking ability and students’ classroom engagement to get a broader understanding of the impact critical thinking has on students’ success. To do this, firstly, both variables of this study are defined and explicated. Then, the relationship between critical thinking and students’ classroom engagement is discussed. Finally, the implications of this research and also its limitations along with suggestions for further studies are put forward.

## Introduction

“Critical thinking enables individuals to use standards of argumentation, rules of logic, standards of practical deliberation, standards governing inquiry and justification in specialized areas of study, standards for judging intellectual products, etc.” (Bailin et al., 1999, p. 291). Paul and Elder (2007) conceptualized critical thinking as the art of analysis and evaluation, considering the point that it can be improved since a quality life needs the quality of thinking. Facione (2011) noted that happiness cannot be guaranteed even if good judgment is practiced and critical thinking is enhanced; however, it undoubtedly offers more opportunities for this goal to be achieved. It has been stressed that autonomy can be shaped through critical thinking ability and one’s learning process can critically be evaluated (Delmastro and Balada, 2012). According to a study conducted by Marin and Pava (2017), English as a foreign language (EFL) critical thinker has the following characteristics: they are active, continuously asking questions, and seeking information which helps them build associations between L2 learning and other features of everyday life. They describe as people, having the capability to analyze and organize thoughts that can be expressed through speaking and writing. They almost always tries to put what has learned before into practice. Beyond doubt, in order to enhance critical thinking skill in EFL learners, teachers should consider the point that teaching is not just about grammar and vocabulary; instead, it concentrates on enhancing teaching, encouraging to be creative, encourage to learn independently, strategies for making decisions and evaluating himself. Similarly, opportunities must be provided by the educators to provide a learning environment in which autonomous learning, active engagement, reflection on learners’ learning process, and L2 advancement are emphasized, for instance, task-based activities. Thus, this study is different from other studies since the focus is placed on teachers’ critical thinking ability to help students thrive rather than students’ critical thinking ability. The reason is that differentiates it from the previous studies is that providing students with opportunities, in which thinking differently is appreciated, would be absolutely rewarding and it is the skill that should be much more highlighted in the studies. Therefore, critical thinking is a skill through which students’ confidence can be raised, leading to their active engagement in the classroom and their being successful since they can see the issues from a different point of view and novel solutions to those problems can be proposed. In the current study, first of all, both teachers’ critical thinking ability and students’ classroom engagement have been discussed. Given that, the association between these two variables has been dealt with. Then, the implications and restrictions of the study as well as some recommendations for further studies have been proposed.

## Background

### Teachers’ Critical Thinking Ability

Critical thinking has attracted much attention since teachers’ way of thinking and beliefs has a pivotal impact on what students achieve in terms of academic success and attainments. Dewey (1933, p. 9), who can be regarded as the father of modern critical thinking, conceptualized it as “active, persistent, and careful of a belief or supposed form of knowledge in the light of the grounds which support it and the further conclusions to which it tends.” As defined by Chance (1986), critical thinking is conceptualized as the capability that one puts into practice to do the followings through this ability: facts which are analyzed, ideas that are generated and organized, opinions that are defended, comparisons that are made, inferences that are drawn, arguments which are evaluated, ideas that are organized, and problems that are solved. As stated by Vdovina and Gaibisso (2013), critical thinking is relevant to quality thinking that enables learners to communicate with others, gain knowledge, and deal with ideas, attitudes, and beliefs in a more skillful way. Based on what has been proposed by Shirkhani and Fahim (2011), critical thinking is an integral factor in many ways. The first reason that can be taken into consideration is that when language learners take responsibility for the way they think; they can evaluate the way they learn in a more successful way. Secondly, critical thinking causes learners to experience a meaningful process of learning in which learning a language is meaningful to them. Thirdly, critical thinking and learners’ achievement are positively correlated. If the learners are shown how to think critically, they get proficient in learning a language. Likewise, Liaw (2007) study indicated that when the content-based approach is implemented in the class, it promotes EFL students’ critical thinking skills. It should be noted that in a content-based approach, attention is focused on the content and what can be perceived through it.

Besides, as Davidson (1998) noted, “the English teachers are expected to provide learners with the ability to communicate with native speakers, valuing overt comments, clever criticism, and intellectual claims.” In a similar manner, Meyers (1986) proposed that teachers can facilitate critical thinking through the activities that are assigned, the tasks that are set, and the feedback that is provided. A study done in a Chinese context by Li and Liu (2021) put forward the taxonomy of critical thinking ability in the EFL learning context and in this study, five skills through which critical thinking can be practiced, were proposed: analyzing, inferring, evaluating, synthesizing, and self-reflection/self-correction (Wang and Derakhshan, 2021). Li (2021) also indicated that the development of critical thinking in international students can be facilitated by learning Chinese. According to a study done by Birjandi and Bagherkazemi (2010), a critical thinker has the following characteristics:

• problems are identified by them and relevant solutions are dealt with,
• valid and invalid inferences are recognized by them,
• decisions and judgments are suspended by them when there is not enough evidence to prove it
• the difference between logical reasoning and justifying is perceived by them
• relevant questions are asked by them to see if their students have understood
• statements and arguments are evaluated
• lack of understanding can be accepted by them
• they have developed a sense of curiosity
• clear criteria for analyzing ideas are defined
• he is a good listener and gives others feedback
• he believes that critical thinking is a never-ending process that needs to be evaluated
• judgment is suspended by them until all facts have been collected
• they seek evidence for the assumptions to be advocated
• opinions are adjusted by them when there are some new facts
• incorrect information is easily rejected by them.

Consequently, according to the characteristics mentioned above, teachers with the ability to think critically is good problem solvers and when facing a problem during the class, they can have greater reasoning skills so as to find a solution to the problem. They are curious and they also ask their students questions to create a sense of curiosity in them. Additionally, they do not accept the new ideas easily, instead, they analyze them and sometimes make them better.

### Classroom Engagement

Engagement is an inseparable part of the learning process and a multifold phenomenon. Classroom engagement refers to the amount of participation that students take in the class to be actively involved in the activities and whether the mental and physical activities have a goal. Engagement itself is a context-oriented phrase which relies on cultures, families, school activities, and peers (Finn and Zimmer, 2012). It has been categorized into different groups: Behavioral engagement such as the amount to which students participate actively in the class; emotional engagement pertains to high levels of enthusiasm which is linked to high levels of boredom and anxiety; cognitive engagement such as the usage of learning strategy and self-regulation; agentic engagement such as the amount of conscious effort so that the learning experience would be enriched (Wang and Guan, 2020; Hiver et al., 2021). Amongst the aforementioned categories, the one which is strongly important in the learning process is behavioral engagement in that it is relevant to the actual recognition of an individual’s learning talents (Dörnyei, 2019). Another possibility that can be viewed is to consider engagement from two other aspects, internal and external. The former implies how much time and effort is allocated to the process of the learning. The latter entails the measures that are taken at the institutional level so that the resources would be dealt with along with other options of learning and services for support, encouraging the involvement in activities leading to the possible outcomes such as consistency and satisfaction (Harper and Quaye, 2009).

Much attention is deserved to be paid to engagement since it is perceived as a behavioral means with which students’ motivation can be realized and as a result, development through the learning process can occur (Jang et al., 2010). Active involvement should be strengthened in L2 classes to prevent disruptive behaviors and diminish the valence of emotions that are negative such as feeling anxious, frustrated, and bored.

Regarding “classroom engagement,” its opposie word “disengagement” can play a significant role in not engaging the students in the class, leading to them feeling bored and demotivated in the class, so from this aspect, it would be worth considering this phrase as well. It has been claimed by some authors (Skinner, 2016) that disengagement itself does not happen frequently in educational settings due largely to the fact that it is related to extreme behaviors, and it is when another phrase disaffection can be considered significant. Disaffection is characterized by disinterest, aversion, resignation, and reduced effort. Therefore, our perception of boredom as a complex emotion can be enhanced, and it can be dealt with more systematically if boredom is viewed through the following factors, disengagement, and disaffection (Wang and Guan, 2020; Derakhshan et al., 2021). As Elder and Paul (2004) mentioned, students should be taught to actively make questions- that is a good emblem of engagement- which is a radical part of critical thinking. The more the students can question, the more they can learn. Some students get accustomed to memorizing the facts and have never been faced with the outcomes of the poor decisions they made since there is always someone to back them and they had better be challenged, being questioned by their teachers (Rezaei et al., 2011).

### The Relationship Between Teachers’ Critical Thinking Ability and Classroom Engagement

Critical thinking has been said to widen one’s horizon because it may shape students’ mindsets and help them take a look at items from a different viewpoint. When one has learned to think critically, they will never accept the *status quo* easily, he will welcome the opposing ideas and will evaluate the arguments. In the EFL context, when a learner has the capability to think critically, or he has been taught to think critically, he always looks for reasons learning new materials and in this respect, his curiosity allows him to learn everything in depth and challenge his schemata to make a link between the newly learned ideas and the ones he has already known. Critical thinking is not a term that can be utilized just for the specific type of people; it can be taught and practiced to be enhanced. The way ideas can be generated and the way comparisons can be made is highly relevant to what has been called critical thinking. Different items can be conceptualized in different ways when we look at them through the lens of critical thinking; therefore, it can have a positive effect on students’ mindsets and the way they live. From an educational point of view, the decisions that have been made by the students, the solutions that have been put forward to tackle a problem when it comes to a learning context, and the way through which their process of learning is ameliorated are all impacted by teachers’ critical thinking. When teachers think critically and they strive to see different skills from a different point of view, it is where students’ sense of curiosity is tickled and their imagination is stretched so as to think of things in a various way.

## Implications and Further Suggestions for Research

Critical thinking is believed to have an enormous effect on students’ classroom engagement. As mentioned above, according to Dewey (1933), the more the students practice thinking critically, the more successful they are in terms of academic achievements because they can decide more rationally, and their problems can be addressed more sensibly. Attention should be paid that this study is of great significance for those people who are engaged in the learning process including those devising curriculums, develop materials, teachers, and learners. Critical thinking is a skill that should be developed in learners so that they would compare and contrast ideas, and as a result, decide wisely and accomplish what they have planned for. Accordingly, opportunities must be provided by the educators to provide a learning environment in which autonomous learning, active engagement, reflection on learners’ learning process, and L2 advancement are emphasized, for example, task-based activities (Han and Wang, 2021).

Additionally, further studies can be done to find more about the variables in this study.

With regard to various age groups, the understanding of critical thinking might be different. Teenagers are said to start thinking critically and hypothetically; however, undoubtedly there is a big difference between what can be perceived about critical thinking by teenagers and adolescents in the educational contexts. Consequently, how different levels of critical thinking can be conceptualized in the learning context is one of the studies that can be conducted in the future. Secondly, teachers’ success and well-being are also tremendously affected by the way they think. Therefore, from this point of view, a study can be conducted in the future so as to find the correlation between teachers’ critical thinking and other aspects of their lives. The reason why this study should be carried out is that considering the L2 environment, students’ way of thinking is impacted by how they are treated by their teachers. Teachers are supposed to equip students with techniques through which the learning process will be facilitated and students’ creativity will be boosted, therefore, it is what helps them to be critical thinkers both in the classroom context and out of it. Another line of research that is worth being done is that diverse activities that can enhance learners’ ability of critical thinking should be categorized based on learners’ characters. In a modern educational world where individual differences are emphasized, classroom activities should be classified, regarding the learning differences of the learners. Therefore, according to Birjandi and Bagherkazemi (2010); Vdovina and Gaibisso (2013), and Li and Liu (2021), teachers’ critical thinking ability play a vital role in how students are engaged in the class.

## Author Contributions

The author confirms being the sole contributor of this work and has approved it for publication.

## Conflict of Interest

The author declares that the research was conducted in the absence of any commercial or financial relationships that could be construed as a potential conflict of interest.

## Publisher’s Note

All claims expressed in this article are solely those of the authors and do not necessarily represent those of their affiliated organizations, or those of the publisher, the editors and the reviewers. Any product that may be evaluated in this article, or claim that may be made by its manufacturer, is not guaranteed or endorsed by the publisher.
